# Low leptin levels are associated with elevated physical activity among lean school children in rural Tanzania

**DOI:** 10.1186/s12889-022-12949-9

**Published:** 2022-05-10

**Authors:** Christine Ludwig, Nadja Knoll-Pientka, Akwilina Mwanri, Celina Erfle, Vincent Onywera, Mark S. Tremblay, Judith Bühlmeier, Agnes Luzak, Maike Ferland, Holger Schulz, Lars Libuda, Johannes Hebebrand

**Affiliations:** 1 Department of Child and Adolescent Psychiatry, Psychosomatics and Psychotherapy, University Hospital Essen, University of Duisburg-Essen, Wickenburgstraße 21, 45147 Essen, Germany; 2 LVR Clinic for Psychosomatic Medicine and Psychotherapy, University of Duisburg-Essen, University Hospital Essen, 45147 Essen, Germany; 3 Department of Food Technology, Nutrition and Consumer Sciences, Sokoine University of Agriculture, P.O. Box 3006, Morogoro, Tanzania; 4 Department of Nutritional Sciences – Professorship of Nutrition in Prevention & Therapy, Justus-Liebig-University Giessen, 35392 Giessen, Germany; 5 Department of Physical Education, Exercise and Sports Science, Kenyatta University, P.O. BOX 43844-00100, Nairobi, Kenya; 6 Healthy Active Living and Obesity Research Group, Children’s Hospital of Eastern Ontario Research Institute, Ottawa, Canada; 7 Institute of Nutrition, Consumption and Health, Faculty of Natural Sciences, Paderborn University, Paderborn, Germany; 8 Institute of Epidemiology, Helmholtz Zentrum München, German Research Center for Environmental Health, Neuherberg, Germany

**Keywords:** Leptin, Physical activity transition, Moderate-to-vigorous physical activity, Sub-Saharan Africa

## Abstract

**Background:**

In Sub-Saharan African countries, rapid urbanization and increasing socio-economic status are associated with a transition to decreased physical activity (PA). A more sedentary lifestyle is linked to increased body fat leading to increments in leptin levels. Since rodent and human studies in high-income countries have shown that starvation-induced hypoleptinemia triggers high PA, efforts are warranted to pursue the hypothesis that low leptin levels in lean children of low- and middle-income countries (LMIC) are also associated with high PA.

**Methods:**

In this cross-sectional study, we assessed seven-day PA with triaxial accelerometry (ActiGraph GT3X) among 223 primary school children (9 to 12 years of age) in rural Tanzania. Moderate-to-vigorous PA (MVPA) and total accelerometer counts per day were outcome variables. Leptin was determined using enzyme linked immunosorbent assay tests from dried blood spots. Anthropometric assessments were conducted and food insecurity and socio-demographic data were gathered using semi-structured interviews.

**Results:**

In this sample of school children in rural Tanzania, leptin concentrations (median: 0.91 ng/mL, P25: 0.55, P75: 1.69), body mass index z-scores (median: -1.35, P25: -1.93, P75: -0.82), and height-for-age-z-scores (median: -1.16, P25: -1.96, P75: -0.61) were low. In contrast, PA levels were high with a median MVPA time of 119 min/day. Linear regression confirmed that leptin levels were negatively associated with MVPA (beta: -18.1; 95%CI: -29.7; -6.5; *p* = 0.002) and total accelerometer counts (beta: -90,256; 95%CI: -154,146; -26,365; *p* = 0.006). Children residing in areas with better infrastructure had lower MVPA levels (*p *< 0.001) and tended to have higher leptin levels (*p* = 0.062) than children residing in areas only reachable via dirt roads.

**Conclusion:**

Our cross-sectional field study is the first that supports the hypothesis of low leptin levels as a potential endocrine trigger of high PA in lean children of a LMIC. We observed early signs of a PA transition towards a less active lifestyle in a subgroup residing in areas with better infrastructure that concomitantly tended to have higher leptin concentrations. Considering that area-dependent PA differences were more pronounced among girls than boys, whereas differences in leptin levels were less pronounced, not only biological, but also external factors explain PA transition.

**Supplementary Information:**

The online version contains supplementary material available at 10.1186/s12889-022-12949-9.

## Background

The exertion of physical activity (PA) depends on various environmental (e.g. type of livelihood, availability of sidewalks and playgrounds) and individual factors (e.g. socio-economic status, biological factors) [1, 2]. During the last decades PA has been susceptible to a secular transition from an active towards a more sedentary lifestyle in many countries [3, 4].

Observations in low- and middle-income countries (LMIC) between 1967 and 2013 have revealed that urbanization and socioeconomic advances were also associated with a trend towards decreasing PA [3]. Such changes are reflected by decreased active transportation, decreased labor-intensive occupational and household work, and increased sedentary activities. As a consequence, known benefits of PA on overall health are likely to diminish [4].

Research using objectively measured, device-based PA among children in countries where PA transition is taking place, such as Sub-Saharan African countries, is scarce [3]. Available studies showed large differences in moderate-to-vigorous PA (MVPA) between countries, year of data collection, and setting [3, 5]. A study among Kenyan school children using pedometers found that 51% of rural children and 26% of urban children achieved a cut-off of 16,500 steps/day [6] providing supporting evidence of a PA transition. Similarly, a systematic review among Sub-Saharan African countries found lower PA levels among children living in urban areas or with a higher socio-economic status (SES) compared to children living in rural areas [3].

Multiple lines of evidence point to a role of the hormone leptin in modulating PA. Leptin, mainly synthesized in adipocytes, exerts multiple functions [7, 8]. One important function is being one of the key modulators in regulating food intake and energy metabolism, by provoking energy expenditure and surpressing food intake via the hypothalamus [9]. During weight maintenance, the most important determinant of circulating leptin concentrations is body fat mass and indirectly body mass index (BMI) [10]. Upon weight loss leptin levels drop and on attainment of sub-physiologic levels (hypoleptinemia) trigger the endocrine adaptation to starvation [11]. Data from rodent studies point to the particular role of hypoleptinemia in relation to high PA levels: in rats, semi-starvation induced hyperactivity entailing increments in running wheel activity of 300–400% can be both prevented and suppressed via exogenous application of leptin [12]. In activity-based anorexia, which is considered a rat model of anorexia nervosa, leptin treatment also reduced running wheel activity [13]. Elevated PA upon food restriction is assumed to represent an adaptive behavior to increase food acquisition [14]. In mice, leptin administration impacted both the motivational and rewarding effects of running through STAT3 signaling in dopamine neurons [15].

Studies on human beings also support the relationship between low leptin levels and elevated PA. In patients with anorexia nervosa, an eating disorder characterized by hypoleptinemia (serum leptin levels < 2 µg/l [16]), hyperactivity of various degrees is frequent [17–19]. An inverted U-shaped relationship between serum leptin levels and PA has been observed [20]. Initial data indicate that the PA drive in these patients decreases upon treatment with metreleptin, a human recombinant leptin analogue [21, 22]. In the latter case study, which also considered device-based assessments, PA decreased in a male anorexia nervosa patient upon metreleptin treatment [22].

Data from high-income countries on an association between circulating leptin and PA among children and adolescents are conflicting. Most cross-sectional studies found a negative association or correlation between leptin and PA among their study participants [23–28], while others found an association only among subgroups, e.g. among girls only [29], or even a positive association in a group of boys with high leptin levels [30]. A longitudinal study among healthy children with normal body weight status from the United Kingdom (UK), found no significant correlation between PA and leptin [31]. Despite these quite comprehensive data from high-income countries, data linking leptin concentrations and PA in children living in LMIC of Sub-Saharan Africa are scarce. If the association between leptin and PA is pronounced in individuals with low leptin concentrations, studies among lean populations such as children of rural areas in LMIC are of special interest. Accordingly, this study aimed to examine the hypothesis that circulating leptin concentrations are inversely associated with device-based measures of PA among lean school children in rural Tanzania, taking potential confounders into account. If correct, increments in circulating leptin upon transition from a rural to an urban lifestyle in LMIC, as a result of improved nutritional status, may represent an endocrine link related to the PA transition. To sample children with low BMI z-scores, who have previously been shown to have the greatest variance of PA [6], we recruited only rural children and preselected those school children with apparently lower body weight.

## Methods

### Study design and schedule

This cross-sectional study was conducted in three wards of Chamwino District, namely Dabalo, Itiso, and Haneti, located in Dodoma Region, United Republic of Tanzania. Data collection took place between May and September 2019 with two breaks due to school holidays. Within each ward, two primary schools were purposively selected based on number of enrolled school children and accessibility. Four primary schools were only reachable via dirt roads. Two primary schools in Haneti were located at the main tarmac road going through Chamwino district. This distinction was used to later examine associations between different infrastructural settings and PA among the study population, with the schools in Haneti considered to have better infrastructure (as a proximal indicator of development). Each school was included consecutively into data collection procedures, which lasted approximately two weeks per school.

At each school, data collection consisted of three parts. The first part included obtaining consent after provision of detailed written and oral information at an information session for parents, establishing screening for participants’ eligibility, taking anthropometric measurements, and blood sampling at school. The second part included device-based assessment of PA of participants for 7 days. During the third part, parents and children were visited at their homestead to collect socio-economic data and information on types of children’s PA.

Ethical approval was obtained from the Ethics Committee of the National Institute for Medical Research, Tanzania and the University of Duisburg-Essen, Germany. Further permission was obtained at the respective regional, district, and ward level. After an information session with parents and children on all study procedures, rights of participants as well as data handling, informed consent and assent forms were signed by all parents of participating children (by signature or fingerprint) and children, respectively. This consent procedure was approved by Ethics Committee of the National Institute for Medical Research, Tanzania. All assessments were conducted in compliance with the Declaration of Helsinki and its later amendments.

### Study population

Our *a priori* sample size calculation was based on an observed correlation between plasma leptin levels and PA among 16 patients with anorexia nervosa of ρ_1_ = -0.410 [20]. In the Tanzanian setting, we expected a weaker correlation of -0.2 between leptin and PA among lean school children. Assuming bivariate normality and a significance level (two-sided) alpha = 0.05, a sample size of 200 children resulted in a power of at least 80%. Considering a dropout rate of at least 10%, we aimed to recruit at least 220 school children.

The recruited study population consisted of a convenience sample. At each school, we aimed to pre-select 15 children (equal sex distribution) from the age groups of 9, 10, 11, and 12 years, respectively. As we expected a higher variance of PA among children in lower BMI ranges, the field staff visually preselected those children at the participating schools with apparently lower body weight and invited their parents to an information session regarding our study. Additionally, we focused on those children who were able to read and write. In some schools, some age groups neither consisted of at least 15 children nor of seven boys or girls. In these cases, all available children within one age group were pre-selected and provided with invitations for their parents. The study’s inclusion criteria were being between 9 to 12 years-old, having no known acute infection or chronic disease, and being able to freely ambulate. After finishing the screening process, a total of 236 children started the study, of whom two were excluded on day two due to withdrawal of consent.

### Anthropometry and blood sampling for leptin measurements

Anthropometric measurements were conducted according to the FANTA protocol [32] and WHO guidelines [33] at each school between 08:00 and 11:15 on two consecutive days. Body weight was assessed once per child using a digital scale (SECA 877, Hamburg, Germany) to the nearest 0.1 kg with children wearing light school uniforms and without shoes. Body height was assessed twice per child to the nearest 0.1 cm with children wearing no shoes using a mobile stadiometer (SECA 217, Hamburg, Germany). The mean of both body height measurements was used for later analysis. BMI z-scores and height-to-age z-scores (HAZ) were calculated using SPSS Macro provided by WHO AnthroPlus software with the WHO Reference 2007 for children 5–19 years [34]. BMI < -2 z-scores was considered as thinness (< -3 z-scores: severe thinness) and HAZ < -2 z-scores was considered as stunting (< -3 z-scores: severe stunting). Mid-upper-arm circumference (MUAC) of the non-dominant arm was measured to the nearest 0.1 cm [32].

Immediately after individual anthropometric measurements, capillary blood samples were taken with disposable one-way lancets from the fingertip. Although not requested, 86% of children reported that they came to school without breakfast. Capillary blood drops were applied onto two circles of filter paper (Whatman 903, SIGMA-ALDRICH, Germany, Fisher Scientific) and stored in a zipper-lock bag (Whatman ZIP Bag 4’ × 6’, SIGMA-ALDRICH, Germany, Fisher Scientific) containing a desiccant, after drying at ambient temperature. Samples were transported at ambient temperature to Benjamin Mkapa Hospital Laboratory in Dodoma on the same day as sampling and stored at -20 °C until transportation to Mediagnost GmbH (Reutlingen, Germany) for leptin analysis in November 2019.

Leptin concentrations were determined in duplicate considering the mean of two quantified leptin concentrations for later data analysis. To extract leptin, four stances per filter paper were obtained using a manual paper punch (Paper Punch Single Hole, 1/8inch, No. 10495010, Schleicher&Schuell, Dassel, Germany), solved with a 250µL dilution (E077 Dilution Buffer, Reagent VP, Mediagnost GmbH, Reutlingen, Germany) and shaken for 12 h (dilution factor 1: 40.58). Afterwards, leptin concentrations were quantified using enzyme linked immunosorbent assay (ELISA) E077 (Sensitive Human Leptin ELISA E077, Mediagnost GmbH, Reutlingen, Germany) at an analytical sensitivity of 0.00117 ng/mL resulting in a lower level of quantification (LoQ) of 0.047 ng/mL. The upper limit of quantification (ULoQ) was 5.113 ng/mL. Quantifications below LoQ (10 measured concentrations from six participants) were set at 0.047 ng/ml, quantifications above the ULoQ (11 measured concentrations from six participants) were set at 5.113 ng/mL.

### Physical activity and ambient temperature

PA data were obtained by ActiGraph GT3X (Pensacola, Florida, USA) accelerometers using ActiLife software (version 6.13.3, firmware v1.9.2). At each school, all participating children, and two designated teachers, received all necessary information on how to wear the accelerometer during an oral session and in written form to take home. Children were instructed to wear the accelerometer, which was attached to an elastic belt, placed at the dominant hip, for seven days from getting up to going to sleep, starting the day after the blood sampling. The accelerometer was removed for water activities as well as for nighttime sleep duration.

Accelerometer data were sampled at 100 Hz, converted to counts and aggregated to 1-min epochs with data filtering set to normal (default recommended set-up from ActiGraph) for further data analysis. Wear time was visually assessed considering the beginning and end of activity measured over the waking period. Days with measured PA during nighttime, e.g., indicating that the sensor was worn after bedtime, were excluded, if no regular pattern of getting up or bedtime could be determined. Based on the NHANES algorithm, non-wear time during the day was classified as an interval of at least 60 min of consecutive zero-counts allowing for up to two consecutive minutes < 100 counts [35]. Days with less than 10 h of defined wear time were excluded and only participants with at least four valid days were considered for analysis [36].

To assign intensity levels, triaxial cut-points for children based on the cut-offs by Romanzini et al. were applied [37], i.e. sedentary (≤ 720 counts/minute), light (> 720 to < 3028 counts/minute), moderate (≥ 3028 to < 4448 counts/minute), and vigorous (≥ 4448 counts/minute). PA counts of ≥ 3028/minute were summarized as MVPA and were considered as the main outcome. For each intensity level, the mean minutes per day per participant were calculated. The mean sum of vector magnitude counts per day was used to describe overall PA as a secondary outcome. Evaluation of adherence to PA recommendations based on the recommended 60 min/day MVPA by WHO [38] and 16,500 steps/day used as cut-off by Onywera et al. [6]. Regarding MVPA, evaluations were based on calculated averaged MVPA over the whole study period.

Information to estimate ambient temperature was retrieved post-hoc from “https://www.worldweatheronline.com” using Dodoma (city) as a proxy location for the study area.

### Information on sample characteristics

During the week of wearing the accelerometers, children and their parents were visited at their homestead and semi-structured interviews were used to collect i) socio-economic data including education and occupation of parents as well as a household’s monthly income, and ii) children’s usual physical activities including time and mode of transport to school. Data were recorded onto tablets and saved onto a computer after each survey day. Three enumerators conducted interviews with parent–child pairs in their national language of *Kiswahili*.

Questions regarding PA were based on WHO Global Physical Activity Questionnaire [39] and KIDS-CAN-questionnaire [6, 40] and were adapted to Tanzanian activities such as collecting firewood or fetching water. Based on this self-reported information, walking time to school was considered as a potential covariate in linear regression models. Food insecurity was assessed by applying the household food insecurity experience scale inquiring on the 30 days preceding the interview. To include this variable into the linear model, the raw score (ranging from 0 to 8) was used to assess food insecurity with higher raw scores corresponding to higher levels of food insecurity [41].

### Statistics

Data analyses were conducted using SPSS Version 25 and SPSS Subscription (IBM SPSS Statistics for Windows, version 25 and SUBCRIPTION, IBM Corp., Armonk, N.Y., USA). Data are presented as median and interquartile range (IQR), as the majority of variables were non-normally distributed, or as frequencies. The two outcome variables, MVPA and PA counts were normally distributed. Sex-differences were tested with Kruskal–Wallis test for non-normally distributed data or t-test for normally distributed data. Correlations between leptin and PA outcomes were calculated with Spearman Rho.

To test our hypothesis of an inverse association between leptin and PA, linear regression analyses were performed with MVPA (model 1) as main outcome and total counts (model 2) as secondary outcome. Before analysis, leptin concentrations were square-root transformed due to their positive skewness. In a second step, potential covariates were tested in these basic models, including sex, age, BMI z-score, walking time to get to school, ambient temperature, and food insecurity. Walking time to get to school was assessed during the interview in five categories (see Table 3) and included as a metric variable in the linear regression model assuming a value of 5 min for category one, 10 min for category two, 22.5 min for category three, 45 min for category four, and 60 min for category five. Inclusion of BMI z-score, instead of MUAC, was chosen based on the association of BMI z-score in the core model. In addition, body weight and height measurements seemed to be more reliable than MUAC measurements. Only those covariates that significantly predicted the respective outcome were finally included in the fully adjusted models, i.e. sex, BMI z-score, walking time to get to school, and ambient temperature. Post-hoc analyses included testing of potential interactions between leptin and MVPA for sex, age, BMI z-score as a continuous variable as well as BMI z-score as a cut-off to obtain two groups (below and above population-specific median) in the basic and adjusted models. A p-value of 0.05 was considered statistically significant.

## Results

### Sample characteristics

**Fig. 1:**
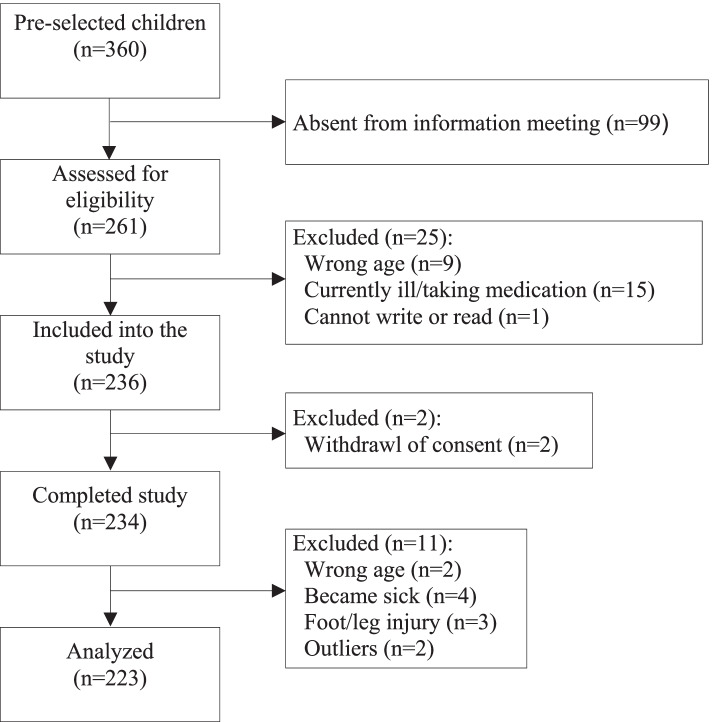
Study flow chart

Of the 236 school children from six primary schools, 234 children completed the study. After data cleaning, data of 223 children (53% girls) were considered for analyses (Fig. 1). Anthropometric data of the children (median age: 11.1 (IQR: 10.2, 11.8) years) indicate that individual demands of energy intake might not have been met in large parts of our sample with median BMI- and height-to-age z-scores both being more than one SD below the median of the WHO reference population [34]. Overall, 37% of children were undernourished, i.e. either too thin according to BMI z-score (< -2 z-scores) or stunted according to HAZ (< -2 z-scores) or both. Corresponding to the low BMI, median leptin concentration was below 1 ng/ml in the total sample with boys having significantly lower concentrations than girls (0.69 ng/mL (IQR): 0.41; 0.96) vs. 1.28 ng/mL (IQR: 0.81; 2.67), *p* < 0.001) (Table 1). Data on SES reflected the rural character of the residence of our sample with the majority of caregivers finishing only primary school and with three-fourths working as farmers (Additional file 1: Table S1).

**Table 1:** Somatic characteristics of study sample of school children in rural central Tanzania (*N* = 223)

Children’s characteristics	Overall (*N* = 223)	Girls (*n* = 118)	Boys (*n* = 105)
Age [years]	11.1 (10.2; 11.8)	11.0 (10.2; 11.6)	11.1 (10.3; 11.9)
Leptin [ng/mL]	0.91 (0.55; 1.69)	1.28 (0.81; 2.67)	0.69 (0.41; 0.96)
Body height [cm]	135.1 (131.4; 140.6)	134.9 (130.1; 140.4)	135.1 (131.6; 141.6)
HAZ	-1.16 (-1.96; -0.61)	-1.34 (-2.16; -0.69)	-1.05 (-1.71; -0.58)
Body weight [kg]	27.3 (25.0; 30.1)	27.1 (24.4; 29.7)	27.6 (25.7; 30.8)
BMI [kg/m^2^]	14.9 (14.1; 15.7)	14.6 (13.8; 15.5)	15.1 (14.5; 15.8)
BMI-z-score	-1.35 (-1.93; -0.82)	-1.49 (-2.03; -0.91)	-1.11 (-1.67; -0.73)
MUAC [cm]	18.1 (17.4; 19.3)	18.2 (17.5; 19.6)	18.1 (17.4; 18.9)
Prevalence of malnutrition* [%]			
Severe thinness	3.1	2.5	3.8
Thinness	17.9	23.7	11.4
Normal weight	78.9	73.7	84.8
Severe stunting	1.3	0.8	1.9
Moderate stunting	22.0	28.8	14.3
Normal height	76.7	70.3	83.8
Undernutrition**	37.3	45.8	28.6

Data presented as medians and interquartile ranges or frequencies; *HAZ* height-for-age z-score, *BMI* body mass index, *MUAC* mid upper arm circumference

*Prevalence of malnutrition based on WHO [34]

**Child is stunted and/or too thin

Generally, PA assessment via accelerometers indicated a high compliance with 90% of children having seven valid wear time days. More than three-fourths of the children wore their accelerometers for at least 12 h per day. Although most time was spent in sedentary activities, a substantial proportion of the time was spent in MVPA (119.4 min/day (IQR: 94.7; 145.0), with the median in boys being approximately 15 min higher than in girls (Table 2) (*p* < 0.001). Higher values in boys than in girls were also observed for total counts of PA/day. Based on the individual averaged MVPA, 93% of children achieved the recommended 60 min/day MVPA [38] (boys: 99% girls: 87%, *p* = 0.001).

**Table 2:** Physical activity (PA) of study sample of school children in rural central Tanzania (*N* = 223)

Physical activity parameters^a^	Overall (*N* = 223)	Girls (*n* = 118)	Boys (*n* = 105)
Moderate-to-vigorous PA [min/day]	119.4 (94.7; 145.0)	105.9 (80.7; 128.7)	131.9 (113.7; 165.7)
Mean total counts/day	1,153,833 (1,012,454; 1,305,158)	1,130,590 (970,892; 1,242,844)	1,219,716 (1,078,160; 1,345,540)
Sedentary PA [min/day]	422.1 (380.1; 466.7)	428.4 (377.7; 461.0)	421.0 (393.0; 469.7)
Light PA [min/day]	315.1 (288.4; 342.6)	328.6 (301.9; 357.1)	302.3 (275.4; 328.3)
Moderate PA [min/day]	73.7 (60.7; 88.0)	68.9 (54.6; 83.7)	79.9 (66.3; 94.1)
Vigorous PA [min/day]	43.1 (27.4; 58.9)	34.0 (21.3; 46.1)	50.9 (39.3; 74.0)
Steps [day]	17,004 (14,736; 19,396)	15,946 (13,730; 17,705)	18,519 (16,438; 20,696)
Wear-time-week [days]	7.0 (7.0; 7.0)	7.0 (7.0; 7.0)	7.0 (7.0; 7.0)
Wear-time-day [min/day]	864.3 (832.3; 892.3)	861.5 (835.3; 887.1)	868. 9 (832.3; 899.6)
**Adherence to WHO recommendations on MVPA**^**b**^** [%]**			
Based on calculated average	93	87	99
At least on 5 days	87	78	97
** Adherence to ≥ 16,500 steps/day**^**c,d**^** [%]**	57	42	73
**Walking distance to school [%]**			
≤ 5 min	11.2	8.5	14.3
> 5 to 15 min	27.4	27.1	27.6
> 15 to 30 min	40.4	43.2	37.1
> 30 to 60 min	15.7	14.4	17.1
> 60 min	5.4	6.8	3.8
**Type of activity [min/day]**			
Screen time: TV and/or video games (*n* = 221)	0.0 (0.0; 12.9)	0.0 (0.0; 4.3)	0.0 (0.0; 17.1)
Time spent reading/doing homework (*n* = 216)	4.3 (0.0; 17.1)	5.0 (0.0; 17.1)	4.3 (0.0; 17.1)
Time spent playing outside (*n* = 220)	54.3 (27.9; 60.0)	42.9 (25.7; 60.0)	60.0 (34.3; 90.0)
Time spent on household chores/farming (*n* = 222)	40.0 821.4; 60.0)	42.9 (22.9; 60.0)	34.3 (21.4; 68.6)

Data presented as medians and interquartile ranges or frequencies;

^a^Cut-off points according to Romanzini et al. [37]

^b^WHO [38]

^c^Cut-off point used as by Onywera et al. [6]

^d^Based on calculated average, *MVPA* moderate-to-vigorous physical activity

Based on questionnaire data, types of PA mainly consisted of playground activities and household chores. Additionally, all children reported using an active mode of transportation to school on most school days with more than 60% having to walk at least 15 min to school. In contrast, overall screen-time was low in this sample with only 38% watching some TV or playing games on their parents’ cell phones (Table 2).

**Fig. 2:**
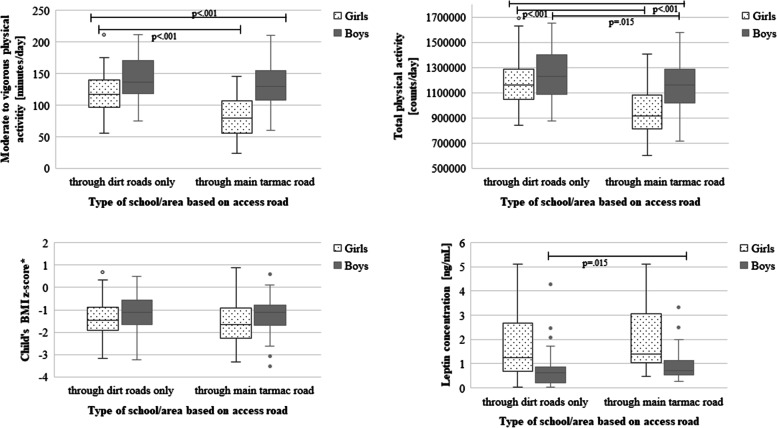
Physical activity parameters, BMI z-score, and leptin concentrations according to infrastructural settings and sex. Differences in moderate to vigorous physical activity, counts, and BMI z-score between infrastructural settings tested by t-test, differences in leptin by Mann-Whitney U-test (only significant p-values included); differences between sexes not depicted. *WHO Reference population [34], *BMI* body mass index

Children living in wards only reachable via dirt roads (Dabalo and Itiso) had higher levels of MVPA (*p* < 0.001), higher total PA (*p* < 0.001), and total steps (*p* = 0.019) than children living in a ward (Haneti) linked to better infrastructure. Although leptin concentrations tended to be lower in children from Dabalo and Itiso than in those children from Haneti (*p* = 0.062, Fig. 2), BMI z-scores of children did not differ between residential areas (mean and standard deviation of BMI z-scores of children of Dabalo and Itiso (*n* = 143): -1.3 ± 0.8 vs. mean and standard deviation of BMI z-scores of children of Haneti (*n* = 80): -1.4 ± 0.9, *p* = 0.487).

### Relationship between leptin concentrations and parameters of PA

Apart from univariate analyses showing inverse correlations between leptin and PA parameters (Additional file 2: Univariate correlations), basic linear regression models confirmed our hypothesis that leptin concentrations were negatively associated with both, MVPA and total counts (Table 3, models 1a and 2a). Leptin accounted for 14% (9%) of the variance of MVPA (total counts) in these models. Adjusting for sex, BMI z-score, time spent walking to school, and ambient temperature in fully adjusted models slightly attenuated the p-values, but associations of leptin concentrations with both parameters remained significant (Table 3, models 1b and 2b). Sensitivity analyses with inclusion of two outliers did not change the negative associations between leptin and the outcomes (data not shown).

**Table 3:** Results of linear model of predictors of physical activity parameters among Tanzanian school children (*N* = 223)*

**Outcome**		**B**	**95% CI**	**SE B**	**β**	**p**
**MVPA**	**Model 1a**^**a**^					
	Constant	152.24	140.52, 163.96	5.95		< .001
	Leptin	-30.26	-40.38, -20.14	5.14	-.37	< .001
	**Model 1b**^**b**^					
	Constant	312.26	214.41, 410.11	49.65		< .001
	Leptin	-18.06	-29.66, -6.47	5.88	-.22	.002
	Male sex	23.77	13.35, 34.19	5.29	.30	< .001
	BMI z-score	5.62	-0.37, 11.62	3.04	.12	.066
	Walking time to get to school	0.64	0.36, 0.93	0.14	.25	< .001
	Ambient temperature	-8.37	-12.77, -3.94	2.25	-.22	< .001
		**B**	**95% CI**	**SE B**	**β**	**p**
**Total counts**	**Model 2a**^**c**^					
	Constant	1,297,387.1	1,234,793.8, 1,359,980.4	31,761.1		< .001
	Leptin	-129,893.9	-183,973.0, -75,814.7	27,440.8	-.30	< .001
	**Model 2b**^**d**^					
	Constant	247,033.8	1,957,655.7, 3,036,412.0	273,663.2		< .001
	Leptin	-90,255.7	-154,146.1, -26,365.3	32,415.9	-.21	.006
	Male sex	67,404.9	9969.2, 124,840.6	29,141.0	.16	.022
	BMI z-score	32,110.3	-932.9, 65,153.4	16,765.1	.13	.057
	Walking time to get to school	2610.2	1045.3, 4175.1	794.0	.20	.001
	Ambient temperature	-56,462.3	-80,858.4, -32,066.2	12,377.8	-.28	< .001

B: unstandardized regression coefficient, β: standardized regression coefficient;

*Model 1a and 2a unadjusted; Model 1b and 2b hierarchical inclusion of other predictors showing significance after inclusion in Model 1/1a;

^a^R^2^=.14; ^b^R^2^=.32, adjusted R^2^=.31 ΔR^2^=.19, ^c^R^2^=.09, ^d^R^2^=.24, ΔR^2^=.15, adjusted R^2^ =.23 (all *p*<.001), *BMI* body mass index.

Sex was observed to be the strongest predictor of MVPA, but post-hoc testing for interaction did not show a significant effect modification in the basic (further including leptin and sex as predictors) and adjusted models. Age and BMI z-score, too, did not reveal significant interactions. Regarding BMI groups (below and above median BMI z-score), the association based on beta values was slightly higher in children with lower BMI (group below median BMI-z-score: beta = -0.429, *p* < 0.001, R^2^ = 0.18, group above median BMI z-score: beta = -0.383, *p* < 0.001, R^2^ = 0.15). In the fully adjusted model, the association with leptin remained significant only in the low BMI group, but not in the high BMI group (group below median BMI z-score: beta = -0.261 *p* = 0.005, R^2^ = 0.29, group above median BMI z-score: beta = -0.107 *p* = 0.280, R^2^ = 0.38).

## Discussion

To the best of our knowledge, this is the first study to examine the association between leptin concentration and PA among lean school children in rural areas of a LMIC of Sub-Saharan Africa. The results of the linear regression analyses supported our hypothesis that low levels of leptin, which can be linked to the low body weight status in our sample, were associated with high PA. This inverse association remained significant after adjusting for walking time to school and other covariates indicating that this relationship seems to be independent from environmental factors and necessary PA. The cross-sectional design of our study does not allow conclusion on cause and effect. Findings from a meta-analysis among obese children showed that exercise intervention significantly decreased leptin levels [42], indicating the leptin changes might be a result of PA. Accordingly, the high PA of the lean children in our study could entail the observed low leptin secretion. Notably, in many of the included studies in the meta-analysis [42], body fat or body weight decreased as well [43–45]. Since the meta-analysis only included obese children and adolescents, a direct comparison to our study participants, characterized by lean body stature, is difficult. Another meta-analysis among a broad range of study participants (children, adolescents, and adults) with various weight categories reported decreasing leptin levels being associated with reduced body fat and weight due to chronic exercise. The decrease in body fat was one of the most significant factors associated with a decrease in leptin levels. Nevertheless, according to their results, chronic exercise training seemed to cause an independent decrease in leptin levels. The effect of PA on leptin levels was stronger in adults than in children [46].

On the other hand, studies in rodents have demonstrated the crucial role of reduced leptin secretion in the development of semi-starvation hyperactivity [12, 15]. In accordance with these studies, case reports of patients with anorexia nervosa provide initial evidence in humans that an alleviation of hypoleptinemia via application of human recombinant leptin reduces the drive for activity and PA [21, 22].This finding together with the well-known urge to move and increased physical activity in patients with anorexia nervosa [47, 48] had led to our focus on lean children. Indeed, the observed correlations between leptin and PA in the current study seemed to be more pronounced in children with lower BMI z-scores, although the interaction term was not significant. However, the missing significance of a negative association in the higher BMI group could also be due to reduced statistical power in this subsample. Considering that children living in settings with better infrastructure had not only lower PA levels, but also tended to have higher leptin levels than children living in more remote settings, our results represent a first step to support the hypothesis that leptin may act as a potential endocrine trigger of reduced PA during PA transition. Notably, a bi-directional relationship for leptin and PA might be possible. Future longitudinal studies are warranted to confirm the causal relationship between leptin concentrations and PA in school children in LMIC.

The observed high rate of undernutrition should be addressed and improved in future public health programs to ensure a proper development of school children living in the study area. These improvements would likely entail an increase of the observed low leptin concentrations. In turn, this increment may improve health outcomes, as leptin does not only play a regulatory role in energy metabolism [49], but also in bone metabolism [50]. However, whether an individual increase in leptin as a result of improved nutritional status might reduce PA intra-individually as a tradeoff remains to be studied longitudinally.

An argument for leptin being a trigger for PA is that our study sample with its low median leptin level had higher objectively measured PA not only compared to high-income countries [30, 51], but also to already more urbanized areas of LMICs in Sub-Saharan Africa [52, 53]. Based on averaged MVPA, 93% of the children met the recommended 60 min/day MVPA compared to 29% of European children and adolescents [51] or 24% of adolescents from Sub-Saharan African countries (although the latter one based on self-reported data) [54]. The active lifestyle of our sample results from an active mode to get to school, playing outside during recess at school or after school, household and farming chores, and very low screen-time. The high level of PA in the current study sample was reflected by the high daily step count (median ca. 17,000 steps). Overall, 57% of children achieved the cut-off of 16,500 steps per day [6], which is comparable to the 51% of children from rural Kenya meeting this threshold and substantially higher compared to the 26% of children from urban Kenya [6].

We assume that the reported lower PA in the other settings was not only a consequence of different environmental settings, but at least in parts also due to higher leptin levels related to higher body fat of such children. In line with this hypothesis, our study also showed that leptin levels tended to be higher and PA was lower in children living in areas with better infrastructure compared to their rural counterparts, although differences in leptin levels reached only borderline statistical significance. Notably, area-dependent differences in PA were more pronounced among girls than boys, whereas differences in leptin levels were less pronounced. Our results confirm PA transition is a multifactorial process, not only explained by biological factors such as leptin, but also by external factors, such as the residential surroundings (Fig. 2).

### Association between leptin and physical activity

The results of our linear regression regarding the inverse relationship between leptin and PA was also seen in mostly cross-sectional studies in high-income countries [23–31]. In a study among nine different European countries (*n* = 902), average PA, vigorous physical activity (VPA), and MVPA were negatively associated with leptin concentrations, also after controlling for various confounders such as sex or total body fat [23]. Another inverse relationship between leptin and PA was found among a group of 640 twelve year old children in France, independent of body fat mass and waist-to-hip ratio [24]. Among another group of French children and adolescents (*n* = 510), leptin was negatively associated with PA in girls only [29]. In a study by Martinez-Gomes et al. among 198 girls, leptin had an inverse relation with VPA [25]. Similar observations were found among 390 Finnish girls and boys [28]. A positive correlation, but only with moderate PA and only in the high leptin sub-group was observed among boys (*n* = 94) [30]. One longitudinal study among children from the UK (*n* = 213), which assessed PA at 5, 6, 7, and 8 years, found no correlation with leptin measured at 8 years [31]. In contrast, one prospective study found that high leptin levels at baseline predicted declining MVPA levels over the course of one year in a sample of minority peripubertal girls (*n* = 50) [55]. In summary, the results of some, but not all studies support our finding of an inverse relationship between leptin levels and PA. Different measures of PA, but also different study sample characteristics may explain different findings in some of the studies. Thus, the inverse relationship may not be discernible if only a small subsample of children within a study sample has a low leptin level or if PA measures have low validity and reliability. In our study of preselected lean children, we were able to detect a significant association between leptin levels and PA despite a rather narrow BMI range and reduced variance of leptin levels (leptin values were within the range expected for age and BMI [56]). The narrow BMI range also underlies the much lower correlation of r = 0.279 between BMI and leptin levels in our sample in comparison to European samples, which included children of a much broader BMI range.

Our hypothesis that an inverse relationship between PA and leptin might particularly be observed in study samples with low leptin levels is further supported by our observation that the association between PA and leptin was more pronounced in children of our sample with lower BMI z-scores. This indicates that the relationship between leptin levels and PA may well be non-linear, although the tested interaction term was not significant. However, we cannot exclude the possibility that the missing significance of a negative association in the higher BMI group may be due to reduced statistical power in this subsample. Further studies are warranted to substantiate that leptin is a trigger of decreased PA during PA transition in lean children in particular. When we take a look at patients with anorexia nervosa in high income countries, a recent study concluded that more than 80% experience an increased drive for activity at the time of their lowest body weight [19]. In contrast, the men who participated in the Minnesota starvation-rehabilitation experiment showed reduced PA levels [57]. These differences may reflect an inverted U-shaped relationship between leptin levels and PA as described previously in patients with anorexia nervosa [20].

In our study the association between leptin and PA parameters in our study was still significant after including the time for walking to school as proximal indicator of necessary PA in the linear regression model. Accordingly, we speculate that in the Tanzanian children, the motivation to be physically active is increased in children with low leptin levels in comparison to children with higher leptin levels. Thus, despite long walks to school, the children were continuously highly active. Based on their results in rodents, Fernandes and coworkers have hypothesized that both motivation to be physically active and endurance are boosted by low leptin levels [15]. In this context, the investigators discuss the relationship between physical fitness and leptin levels in marathon runners. In 36 male runners, leptin levels determined two days prior to participation in a marathon were positively correlated with the training status and achieved marathon time. The correlation with the achieved time remained significant after adjustment for age and BMI [58]. Whereas the high levels of PA observed in the Tanzanian children appear favorable in medical terms with an exceedingly high adherence rate of 93% to WHO recommendations on MVPA, the high rates of thinness and stunting suggest a trade-off. Low leptin levels have been associated with reduced immunity and reduced bone growth, which represent common medical problems among children with substantially reduced fat mass [59].

In our sample, we did not observe indications for differing effects of leptin between boys and girls, since their beta estimates were similar. However, PA was higher in boys than in girls. Sex-differences with higher PA levels in males are well known among youth [60] and have also been observed in a review including data from Sub-Saharan African countries [3]. Leptin levels twice as high in girls than in boys of our study sample might depict one biological component of observed lower PA in girls. However, other, gender-specific determinants might also play a role regarding differences in PA (e.g. gender-specific chores), since sex was still a significant predictor of PA in regression models including leptin as explanatory variable.

### Other determinants of physical activity

Next to the observed sex difference in PA, there were several other factors associated with PA such as ambient temperature, walking time to school, and infrastructure. Particularly in the two remote wards self-reported “walking time to get to school” (i.e. active transportation) plays an important role for an active lifestyle. Living in wards with better infrastructure (i.e. paved roads) was associated with being less physically active, which is in line with results from other studies [3, 6, 52]. In contrast, age was not a significant determinant of PA probably due to the rather homogenous and narrow age of the study sample of primary school children. Nevertheless, research has shown decreased PA with increasing age during youth [51].

### Strengths and limitations

To best of our knowledge, the current study is the first that investigated the relationship between leptin and PA in lean school children in a LMIC in Sub-Saharan Africa. Further strengths are a homogenous and sex-balanced study sample, device-based assessments of PA, and high compliance among school children. Analysis using uniaxial counts and cut-offs for MVPA (data not shown) supported the results based on triaxial accelerometry confirming the robustness of our results.

However, due to the recruitment of a convenience sample with focus on inclusion of children with lower weight status, selection bias cannot be ruled out. To minimize confounding, we opted to examine the association between leptin and PA in a relatively homogenous and rural study population. Further, we preselected those with apparently low body weight due to the large variance in PA observed in this subgroup. However, this preselection reduces the external validity of our findings. To better depict possible PA transition among school children and examine its biological and non-biological predictors, inclusion of urban schools should be considered for future studies. Notably, in our study, leptin concentrations were slightly lower than BMI and sex-specific reference values (provided by Mediagnost GmbH [61]). Differences in body fat could underlie this observation. Alternatively, this might also be due to the dried blood spot method used in our setting as compared to venous blood sampling used for the reference values. To fully cover PA among school children, a selection of a representative population-based sample would be ideal. However, data on household size, occupation and education among parents (Table 1) were similar to national data from Tanzania [62]. In addition, household food insecurity (Table 1) was similar to the 63% observed in East Africa in 2018 [63].

Due to the cross-sectional design of the study, firm conclusions on causal effects of leptin concentrations on PA cannot be drawn. Reverse causation cannot be ruled out, i.e. that changes in PA affect leptin levels (via changes in body (fat) composition or other mechanisms). However, evidence in both rodent [12, 13, 15] and human (patients with anorexia nervosa [16, 21, 22]) studies led us to primarily discuss the potential causal role of low leptin levels in high PA. In our study, we did not assess body composition per se, but rather BMI and MUAC. Body fat might have been a better variable that explains variations in leptin, but reliable electronic measurements of body fat or body composition were not feasible in this rural setting. However, other studies showed a significant association between leptin and PA even after adjusting for body fat [24, 25]. In addition, we did not assess pubertal stage among the study participants for reasons of compliance but limited the age range to 9 to 12 years to obtain a homogenous sample regarding puberty. A study addressing age at menarche among girls in Tanzania found a mean age of 14.3 ± 1.1 years [64]. Accordingly, we assume that most children or our sample were prepubertal or at an early stage of puberty.

Quantification of leptin relied upon dried blood spots instead of serum blood samples which might be interpreted as a limitation. However, we chose this technique as it is not as invasive and scary for children as venipuncture and suitable for rural settings. This approach may well have contributed to the high participation rate. In addition, internal data from Mediagnost GmbH demonstrated good validity between different sampling materials.

## Conclusions

To our knowledge, this was the first study to examine a possible link between leptin and PA among primary school children in central, rural Tanzania, with an observed negative association between leptin concentration and PA. Our results may indicate an endocrine pathway affecting PA levels and already showed first signs of a PA transition in that area in children with higher leptin levels. The observed low leptin concentrations, low BMI and height-to-age z-scores among the study population depict nutritional deficits. Although our study sample was not representative for that area, these nutritional deficits and their effect on PA should be further addressed. Since improvements of nutrition status could result in increases of the low leptin levels observed in our study sample, the role of leptin, especially upon transition from (sub-physiologically) low to normal concentrations remains to be analyzed, ideally in longitudinal studies to prove the intraindividual impact of leptin on PA. In such studies, the soluble leptin receptor should be ideally assessed to allow determination of the fraction of biologically active free serum leptin [65]. Further, different leptin receptor genotypes and their leptin sensitivity might be considered (66, 67).

## Supplementary Information

**Additional file 1.**

**Additional file 2.**

## Data Availability

The datasets generated and analyzed during the current study are not publicly available due to the vulnerably characteristic of the study population but are available from the corresponding author on reasonable request.

## Abbreviations

BMI: Body mass index
ELISA: Enzyme linked immunosorbent assay
HAZ: Height-to-age z-score
IQR: Interquartile range
LMIC: Low- and middle-income countries
LoQ: Lower level of quantification
MUAC: Mid upper arm circumference
MVPA: Moderate-to vigorous physical activity
NHANES: National Health and Nutrition Examination Survey
PA: Physical activity
SES: Socio-economic status
STAT3: Signal transducer and activator of transcription 3
TZS: Tanzanian Shilling
ULoQ: Upper level of quantification
VPA: Vigorous physical activity
WHO: World Health Organization
