# Supplementary material for: Low leptin levels are associated with elevated physical activity among lean school children in rural Tanzania
Source: BMC Public Health. 2022 May 10;22:933. doi: 10.1186/s12889-022-12949-9 (PMC9087976; doi:10.1186/s12889-022-12949-9)
Supplement: Supplementary file 1 — Additional file 1. [file 12889_2022_12949_MOESM1_ESM.docx]

## Additional file 1_Table S1. Socio-economic and demographic information based on interviews with caregivers of Tanzanian rural school children (N=223)

| Sex of interviewed caregiver [%] | Overall (N=223) | | |
| --- | --- | --- | --- |
| Female | 71.3 | | |
| Male | 16.6 | | |
| Both were interviewed | 12.1 | | |
| Marital status of caregiver [%] |  | | |
| Single | 4.9 | | |
| Married/living together | 70.4 | | |
| Separated/divorced | 17.5 | | |
| Widowed | 7.2 | | |
| Household size [median (IQR)] | 6.0 (5.0; 7.0) | | |
| Household income per month [%] | | | |
| < 50,000 TZS | 6.7 | | |
| 50,000 – 99,999 TZS | 26.9 | | |
| 100,000 – 149,999 TZS | 18.4 | | |
| 150,000 – 299,999 TZS | 34.5 | | |
| 300,000 – 499,999 TZS | 7.6 | | |
| > 500,000 TZS | 3.1 | | |
| Don’t know/refused to answer | 2.7 | | |
| Educational status [%] | **Maternal (n=221)** | **Paternal (n=175)** | |
| No education | 22.7 | 13.7 | |
| Few years primary | 11.8 | 12.0 | |
| Completed primary | 58.8 | 65.1 | |
| Few years secondary | 4.3 | 2.9 | |
| Completed secondary/higher | 2.4 | 6.3 | |
| Main occupation [%] | **Maternal (n=221)** | **Paternal (n=175)** | |
| Farmer | 75.9 | 75.2 | |
| Pastoralist | 0.0 | 5.8 | |
| Business sector | 10.4 | 5.2 | |
| Housewife | 7.6 | 0.0 | |
| Carpenter | 0.0 | 4.6 | |
| Other | 6.1 | 9.2 | |
| Household food insecurity experience scale [%] | | | **Overall (N=223)** |
| Food secure | 8.1 | | |
| Mildly food insecure | 30.0 | | |
| Moderately food insecure | 39.9 | | |
| Severely food insecure | 22.0 | | |
| Mean ambient temperature during assessment* [°C] | | | |
| School 1 (May 2019) | 22.8 | | |
| School 2 (July 2019) | 21.0 | | |
| School 3 (July/August 2019) | 22.7 | | |
| School 4 (August 2019) | 22.1 | | |
| School 5 (August 2019) | 23.4 | | |
| School 6 (September 2019) | 24.5 | | |

IQR: Interquartile range, TZS: Tanzanian Shilling
