# Supplementary material for: Low leptin levels are associated with elevated physical activity among lean school children in rural Tanzania
Source: BMC Public Health. 2022 May 10;22:933. doi: 10.1186/s12889-022-12949-9 (PMC9087976; doi:10.1186/s12889-022-12949-9)
Supplement: Supplementary file 2 — Additional file 2. [file 12889_2022_12949_MOESM2_ESM.docx]

**Additional file 2_Univariate correlations**

According to univariate analyses using Spearman-Rho, correlations between leptin concentrations and parameters of PA increased with intensity levels of the latter from light PA (r_s_ =.148, p=.05), over moderate activity (r_s_ =-.242, p=.01), towards vigorous activity (r_s_ =-.401, p=.01). Further, leptin was negatively correlated with MVPA (r_s_ =-.351, p=.01), total counts (r_s_=-.260, p=.01), and steps (r_s_=-.313, p=.01). Additionally, leptin concentrations were positively correlated with BMI z-score (r_s_=.279, p=.01) and MUAC (r_s_ =.490, p=.01).
